# ScRNA-Seq and BCR Analysis of Murine Immune Responses to Inactivated DHAV-1 as a Model Antigen

**DOI:** 10.3390/v18040448

**Published:** 2026-04-08

**Authors:** Yaru Fan, Saisai Zhao, Yafei Qin, Guocheng Liu, Linyu Cui, Siming Zhu, Youxiang Diao, Dalin He, Yi Tang

**Affiliations:** 1 College of Veterinary Medicine, Shandong Agricultural University, Tai’an 271018, China; fyr19860911398@163.com (Y.F.); 18754297793@163.com (S.Z.); qyf15082973661@163.com (Y.Q.); 13854082576@163.com (G.L.); cuilinyu2022@163.com (L.C.); 18853857893@163.com (S.Z.); yxdiao@126.com (Y.D.); 2 Institute of Animal Science, Chinese Academy of Agricultural Sciences, 2, Yuanmingyuan West Road, Beijing 100091, China

**Keywords:** DHAV-1, B-cell receptor, scRNA-seq, immunological characteristics

## Abstract

Currently, the B-cell response patterns induced by viral antigens in avian disease models and their detailed immunological characteristics still require comprehensive elucidation at the single-cell level. In this study, we employed single-cell sequencing (scRNA-seq) and B cell library technology to conduct an in-depth analysis of B cells in the spleens of mice with inactivated duck hepatitis A virus type 1 (DHAV-1) as model antigen. This study aimed to investigate the immunological characteristics of the virus antigen in the mouse model and characteristics of B-Cell Receptors. The results showed that the DHAV-1 group had distinct changes in splenic B cell subset counts, proportions, and intercellular communication. Additionally, an increased trend in communication strength between Gm26917+B and Gm11837+B cells was observed, with enriched expression of C-X-C motif chemokine ligand (CXCL) and lymphotoxin (LT) detected in the DHAV-1 group. Furthermore, the DHAV-1 group exhibited a prominent combination of the IGHV1 family and IGHV3-1/IGHJ3 in the heavy (H) chain variable region. Compared with the CK group (negative control group), the amino acid sequence length and diversity of the CDR3 region in the DHAV-1 group exhibited a decreasing trend. In summary, our findings characterize the immunological features of splenic B cells in mice after immunization with inactivated DHAV-1, and provide a preliminary characterization of DHAV-1-induced B cell transcriptional states and BCR repertoire features, generating testable hypotheses for subsequent mechanistic investigations of B cell-mediated immune responses to viral antigens.

## 1. Introduction

Inactivated virus antigens, owing to their stable immunogenicity and well-defined antigenic properties, are widely used as model substances for exploring the fundamental mechanisms of humoral immunity. Duck viral hepatitis (DVH) is caused by the duck hepatitis A virus (DHAV), which belongs to the genus Avian hepatitis virus within the family Picornaviridae [1]. It primarily infects ducklings aged 1–3 weeks. DHAV is classified into three serotypes based on the nucleotide sequences of the viral strains. Phylogenetic analysis based on VP1 sequences indicated that DHAV-1, DHAV-2, and DHAV-3 correspond to serotype I, the Taiwan novel strain, and the Korea novel strain, respectively [2]. Currently, the production of traditional attenuated vaccines and inactivated vaccines using egg-based systems remains the primary and most practical approach [3]. Inactivated DHAV-1, with its well-defined molecular characteristics and ability to induce potent humoral immunity in experimental animals, provides an ideal model for studying B-cell-mediated immune responses. Although DHAV-1 vaccines have demonstrated efficacy in controlling DHAV-1 infection, the specific molecular mechanisms underlying their induction of B-cell immune responses—particularly the dynamic changes and immunological characteristics of the B-cell receptor (BCR)—remain poorly understood. This includes the single-cell resolution dynamics of B-cell subsets, intercellular communication networks, and the BCR repertoire remodeling induced by inactivated DHAV-1. Furthermore, while mouse models offer clear genetic backgrounds and mature single-cell sequencing and BCR library technologies, the corresponding techniques for duckling models are far less developed. Therefore, this study employs mice as immunized animals to investigate the specific patterns of B-cell responses induced by inactivated avian virus antigens.

Single-cell RNA-sequencing (scRNA-seq) technology and related bioinformatics methods have advanced rapidly. Tang et al. pioneered droplet-based scRNA-seq, marking a significant milestone in single-cell analysis [4]. ScRNA-seq has been extensively applied in mouse models to explore developmental biology and disease mechanisms, yielding comprehensive analytical approaches for intercellular interactions and communication during viral infection [5,6,7]. These studies have illuminated neurodevelopment [8,9], cancer [10], tumor microenvironment [11], and inflammatory responses [12]. These findings have advanced virology research and immunotherapy strategies. Previous studies have employed single-cell sequencing to investigate DHAV-3-related mechanisms [13,14]. However, research on the specific molecular mechanisms and cellular heterogeneity of B-cell immune responses following avian viral infections remains relatively limited.

The B cell receptor (BCR) is a membrane-bound immunoglobulin (mIg) typically composed of an antigen-binding subunit and a signaling unit. The antigen-binding subunit comprises two IgHs and two IgLs and features a unique binding site for antigenic determinants. As central participants in humoral immunity, B cells undergo precise regulation of multiple signaling pathways during their activation, proliferation, and differentiation into plasma cells and memory B cells following viral infection. Current research continues to explore the mechanisms of disease onset and immune responses associated with BCR in various viral diseases, such as dengue virus [15], novel coronavirus (COVID-19) [16], herpesvirus [17], pseudorabies virus [18], and other viral diseases. However, studies on waterfowl viruses are scarce. These investigations deepen our understanding of humoral immune mechanisms and responses, providing theoretical support for BCR’s clinical application of BCR.

This study established a mouse model immunized with inactivated DHAV-1 as the model viral antigen and employed single-cell sequencing to conduct an in-depth analysis of splenic B cells, elucidating the intercellular communication networks and interactions among immune cells. Furthermore, it provided an analysis of the pairing of H and L chains, as well as the amino acid length and diversity in the CDR3 region. These findings not only shed light on the pivotal role of B cells in the DHAV-1 immune response but also provide novel insights and an experimental foundation for deepening our understanding of the immune mechanisms underlying viral diseases in waterfowl. Since mice are not natural hosts for DHAV-1, the results of this model reflect only the B-cell immune response to inactivated DHAV-1 antigens in mammals and cannot be directly extrapolated to the natural infection process of DHAV-1 in ducks. By establishing this model and integrating single-cell sequencing technology, we can precisely capture the dynamic changes in B cells during the immune process. This enables us to propose hypotheses regarding the molecular mechanisms underlying B cell responses to avian viral antigens and establishes a preliminary framework for subsequent studies in natural duck hosts.

## 2. Materials and Methods

### 2.1. Virus and Experimental Materials

The DHAV-1 strain used in this study was provided by the Institute of Avian Diseases, Shandong Agricultural University. A formaldehyde solution was purchased from Laiyang Kangde Chemical Co., Ltd. (Laiyang, China). Freund’s complete adjuvant (Cat. No. F5881) and Freund’s incomplete adjuvant (Cat. No. F5506) were purchased from Sigma-Aldrich (St. Louis, MO, USA). Chromium Next GEM Single-Cell 3′ GEM, Library, and Gel Bead Kit v3.1 (Cat. No. PN-1000121) was purchased from the Chromium Company (Pleasanton, CA, USA). Qubit dsDNA Assay Kit (Cat. No. Q328520), and SPRIselect Kit (No. B23318), and DynaBeads^®^ MyOne™ Silanized Magnetic Beads* (Cat. No. 37002D) were purchased from Thermo Fisher Scientific (Waltham, MA, USA). Agilent High-Sensitivity DNA Kit (Cat. No. 5067-4626) was purchased from the manufacturer Agilent (Santa Clara, CA, USA). 10× Genomics Chromium™ Single-Cell V(D)J Enrichment Kit (Cat. No. 1000072) was purchased from 10× Genomics (Pleasanton, CA, USA). EasySep™ Mouse Pan-B Cell Isolation Kit (Cat. No. 19844) was purchased from Vancouver Stem Cell Technology (Vancouver, BC, Canada).

### 2.2. Experimental Animals and Ethics Statement

The BALB/c mice used in this study were purchased from Pengyue Laboratory Animal Breeding Co., Ltd. (Jinan, China). They were housed individually in standard cages under controlled environmental conditions, with feed and bedding provided. The experimental protocol was approved by the Ethical Committee for animals in research of the Shandong Agricultural University, China, and adhered to the ARRIVE guidelines [14].

### 2.3. Experimental Immunization Protocol

The virus was inactivated using a 0.1% (*v*/*v*) formaldehyde solution and incubated at 37 °C for 24 h. Twelve 8-week-old female BALB/c mice were randomly divided into an experimental group (DHAV-1) and a control group (CK), with six mice in each group. For the DHAV-1 group, the immunogen was prepared by emulsifying and mixing the inactivated virus suspension with Freund’s adjuvant at a 1:1 ratio. Primary immunization was performed on day 0 with Freund’s Complete Adjuvant (FCA), followed by booster immunizations on days 14 and 21 with Freund’s Incomplete Adjuvant (FIA). For the CK group, phosphate-buffered saline (PBS) was used as a substitute for the inactivated virus suspension, and the PBS was emulsified with Freund’s adjuvant prior to immunization. Serum antibody titers in the experimental group were measured at each blood collection time point using indirect ELISA. Serum antibody titers peaked on day 28. The mice were euthanized by cervical dislocation, and spleen samples were collected for subsequent analysis [14].

Due to the high cost of the 10× Genomics platform and the limited sample size in this study, the broad representativeness of the data may be compromised. To address this, we meticulously designed our experimental protocol. Spleen B cells from the same group were pooled and simultaneously used for single-cell RNA sequencing and BCR library construction. All samples underwent batch processing and sequencing to minimize systematic variations between samples, thereby enhancing the consistency and stability of the experimental results.

### 2.4. Single-Cell Library Preparation and Sequencing

Spleens were sterilized by immersion in 75% ethanol before preparing a single-cell suspension of splenic cells [19]. B cell isolation via magnetic bead sorting was performed using the EasySep™ Mouse Total B Cell Isolation Kit. Briefly, splenocytes were resuspended in RPMI-1640 medium, and the final volume was adjusted to 0.25–2 mL in a 5 mL tube. A pre-prepared sorting cocktail was added to the cell suspension at a final concentration of 50 µL per mL, followed by thorough vortex mixing and incubation at room temperature for 10 min. Subsequently, 75 µL per mL of RapidSpheres™ magnetic beads was added, mixed well, and the mixture was incubated at room temperature for 2.5 min. RPMI-1640 medium was added to the tube to a total volume of 2.5 mL, and the cell suspension was gently pipetted up and down two to three times. The tube was then placed in a magnet (without a cap) and incubated at room temperature for 2.5 min. Finally, the magnet and tube were inverted continuously to decant the enriched B cell suspension into a new tube. The prepared single-cell suspension was subjected to microscopic counting and quality control assessments, including clumping and fragmentation rates [14].

The cell suspension was adjusted to an optimal concentration for immediate loading onto the 10× Genomics microfluidic platform. 5′ mRNA detection was performed using the Chromium Next GEM 5′ Kit. Beads carrying 10× Barcode, UMI, and Switch Oligo (TSO) were encapsulated with cells within oil droplets to form Gemstone Emulsion Microdroplets (GEMs). After collecting the cell-enclosed droplets, the cells were lysed to release mRNA. The polyA tail was captured by poly-dT in the reaction system. Reverse transcriptase synthesizes cDNA by appending three CCC bases to the 5′ end. This sequence then complements the GGG sequence at the TSO primer end on the Gel Beads, enabling template conversion and transcript extension to capture the 5′ end of the mRNA. Following oil phase separation, cDNA enrichment, and amplification, a full-length cDNA library was formed. Subsequently, the cDNA undergoes enzymatic fragmentation, end repair and A-tailing, ligation, adapter addition, sample indexing, and PCR amplification to construct a DNA sequencing library for high-throughput sequencing. Using the full-length cDNA obtained above, the 10× Genomics TCR/BCR Enrichment and Amplification Kit was used to amplify full-length V(D)J fragments using BCR constant region primers. Corresponding BCR libraries were constructed and subjected to high-throughput sequencing (HTS) [14].

### 2.5. Data Analysis

Basic quality control of the samples was performed using the official 10× Genomics software Cell Ranger (version 7.0.1) [20]. Further data quality control and optimization were performed using Seurat software (version 4.0.0) [20]. Following data refinement, highly variable genes were identified using the FindVariableGenes function within the Seurat package, which enabled dimensionality reduction and clustering analysis. This provides high-quality data for subsequent analyses. Subsequently, marker genes were identified and annotated using the FindAllMarkers function of the Seurat package. Cell type identification and systematic annotation were performed using the SingleR package (version 1.4.1) in conjunction with public reference datasets [21]. Differentially expressed genes were screened using the FindMarkers function in the Seurat package, and enrichment analysis was performed on the screened genes. Additionally, SCENIC analysis was performed using the RcisTarget motif database and GRNboost (SCENIC version 1.2.4, RcisTarget version 1.10.0, and AUCell version 1.12.0) to construct and interpret gene regulatory networks [22]. CellPhoneDB (version 4.1.0) was used to identify ligand-receptor interaction pairs within single-cell transcriptomic data [23]. The GSVA package (version 1.30.0) was used to calculate the pathway activity scores for individual cells. Combined with the LIMMA package (version 3.38.3), it enabled the analysis of differential pathway activity patterns between different groups, completing the GSVA enrichment analysis [24]. Finally, the CellChat R package (version 1.1.3) was used for the systematic analysis of ligand-receptor interactions [14,25].

## 3. Results

### 3.1. Single-Cell Transcriptome Analysis

**Figure 1:**
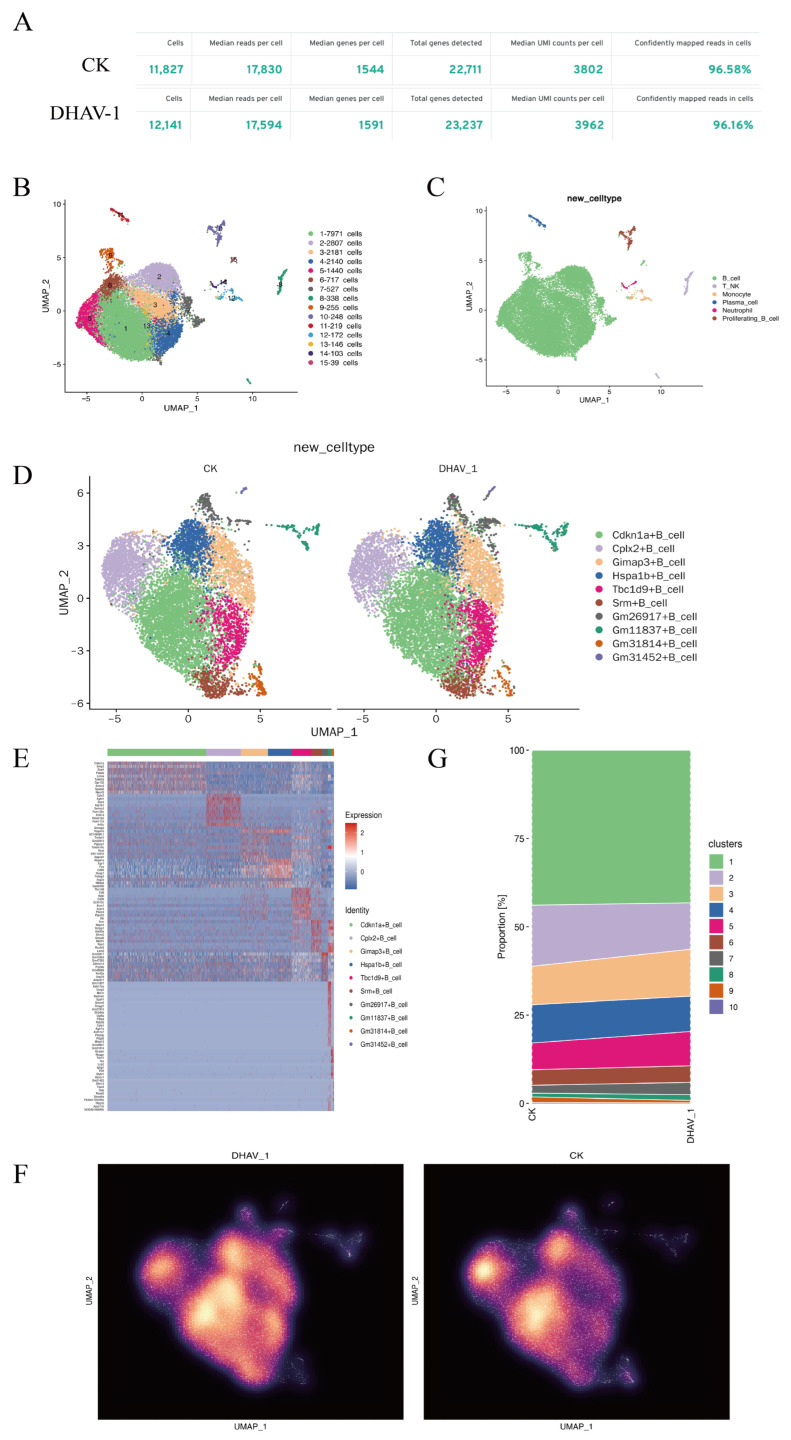
The results of molecular and cellular analyses following sample processing. (**A**) Sample cell quality statistics. (**B**) Visualization of single-cell clusters via UMAP based on PCA-derived dimensionality reduction, with cells from different clusters distinguished by their color. (**C**) Visualization of single-cell clusters, with distinct cell types distinguished by different colors. (**D**) UMAP analysis of cell clusters between the CK and DHAV-1 groups and identification of key molecular markers. (**E**) Heatmap of the expression of marker genes. (**F**) Density plot of UMAP distributions for the DHAV-1 and CK groups. (**G**) Bar chart showing the proportion of cell clusters in the samples (the proportion of cell clusters is described as a trend without statistical significance).

As shown in Figure 1A, following preliminary quality control of the samples, the CK group yielded 11,827 cells, with a median read count per cell of 17,830. The median number of genes per cell was 1544, with a total of 22,711 detected genes. The median number of UMIs per cell was 3802, and 96.58% of the reads were assigned to cells with high confidence. The DHAV-1 group yielded 12,141 cells, with a median of 17,594 reads/cell. The median number of genes per cell was 1591, with a total of 23,237 detected genes. The median number of UMIs per cell was 3962, and 96.16% of the reads were assigned to cells with high confidence. In summary, these results validate the reliability of the single-cell RNA sequencing dataset. These findings establish a solid foundation for subsequent cell type annotation, differential expression analysis, and studies on immune cell interactions between the CK and DHAV-1 groups.

Based on the gene expression profiles per cell, cells with similar expression patterns were clustered into 15 clusters (Figure 1B). Characterized by signature gene expression profiles, these clusters included B_ cells, T_NK, Proliferating_B_ cells, Plasma_ cells, Monocytes, and Neutrophils (Figure 1C). Ten distinct B cell subpopulations were identified (Figure 1D), with each subpopulation named based on the most frequently expressed gene fragment. The Cdkn1a+ B cell subpopulation exhibited the highest expression levels (Figure 1E), and the functional response of B cells in the DHAV-1 group was enhanced compared to that in the CK group (Figure 1F). CdKn1a plays a crucial role in regulating cell cycle arrest during immune responses, balancing innate immunity and inflammation, and participating in antiviral signaling pathways [26]. Through dimensionality reduction and clustering analysis, the DHAV-1 group exhibited a higher proportion of clustering of B cells, proliferating B cells, and monocytes than the CK group (Figure 1G). This indicates that these cell populations may play a key role in mediating immune responses.

### 3.2. The Role of B Cells in Immunity

**Figure 2:**
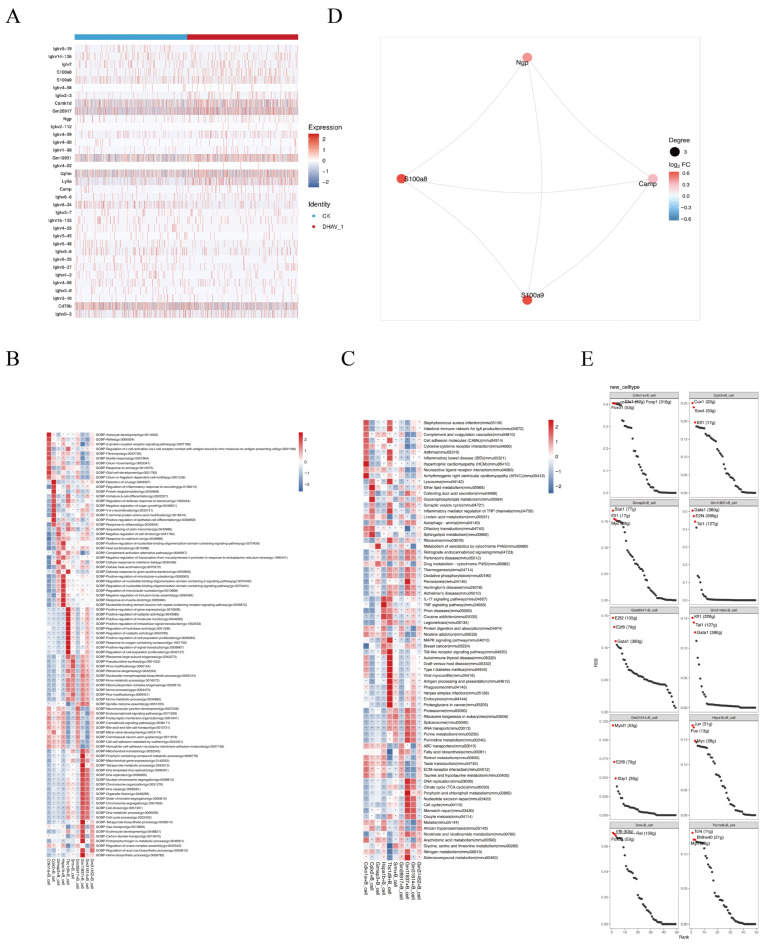
Multi-omics DEGs Data Analysis Results. (**A**) Heatmap of the expression of marker genes. (**B**) Display of GO enrichment results (“*” Indicates statistical significance). (**C**) KEGG enrichment results (“*” Indicates statistical significance). (**D**) Circular plot of differentially expressed gene interactions. (**E**) Ranking chart of regulatory factor specificity.

Analysis of differentially expressed genes (DEGs) distribution across experimental groups (Figure 2A) revealed that when sorted by fold change from highest to lowest, 20 up-regulated and 20 down-regulated genes were selected for heatmap visualization. Based on fold change results, 20 up-regulated and 15 down-regulated genes were identified, totaling 35 differentially expressed genes primarily involved in immunoglobulin production. These genes are associated with antigen recognition, inflammatory responses, and nervous system function. Among these, several genes associated with elevated immunoglobulin levels may provide candidate markers for subsequent neutralizing antibody-related studies. Further GO analysis (Figure 2B) revealed that the differentially expressed genes were primarily enriched in immune system-related processes. KEGG pathway analysis (Figure 2C) indicated that the DEGs in B cells were primarily enriched in pathways related to immune response (e.g., cytokine-cytokine receptor interaction) and cell cycle regulation. These enriched pathways may be involved in the activation, proliferation, and functional differentiation of B cells induced by inactivated DHAV-1 antigen, thereby modulating the host’s humoral immune response to viral antigens.

Gene-protein interaction network analysis (Figure 2D) revealed that S100a8, NGP, CAMP, and S100a9 were all upregulated genes, with interactions between each of the four genes. S100a8 and S100a9 belong to the S100 calcium-binding protein family. The proteins they encode are expressed in various cell types, particularly in immune cells, such as neutrophils and macrophages. The primary function of S100a8 is to regulate intracellular calcium signaling. Calmodulin binds to receptors on the surface of immune cells, triggering signaling pathways that promote the recruitment and activation of these cells [27]. S100a9 is primarily involved in immune responses, inflammatory regulation, and cellular signaling. NGP belongs to the cystatin family. Although this superfamily is closely associated with cancer biology and adaptive immunity, the relationship between macrophage NGP and inflammation and phagocytosis is poorly understood. The CAMP gene encodes proteins capable of directly killing bacteria, fungi, and certain viruses, while also modulating immune responses by binding to receptors on immune cells. Gene-protein interaction networks play crucial roles in various inflammatory responses and immune activities. During B cell development, diverse transcription factors exert significant effects at multiple stages, including cell proliferation and differentiation (e.g., CUX1, E2F2, MYBL1, and JUN), metabolism, and immune responses (e.g., SOX4, STAT1, and TCF4) (Figure 2E).

### 3.3. Potential Cellular Communication Mechanisms of B Cells in Immune Processes

**Figure 3:**
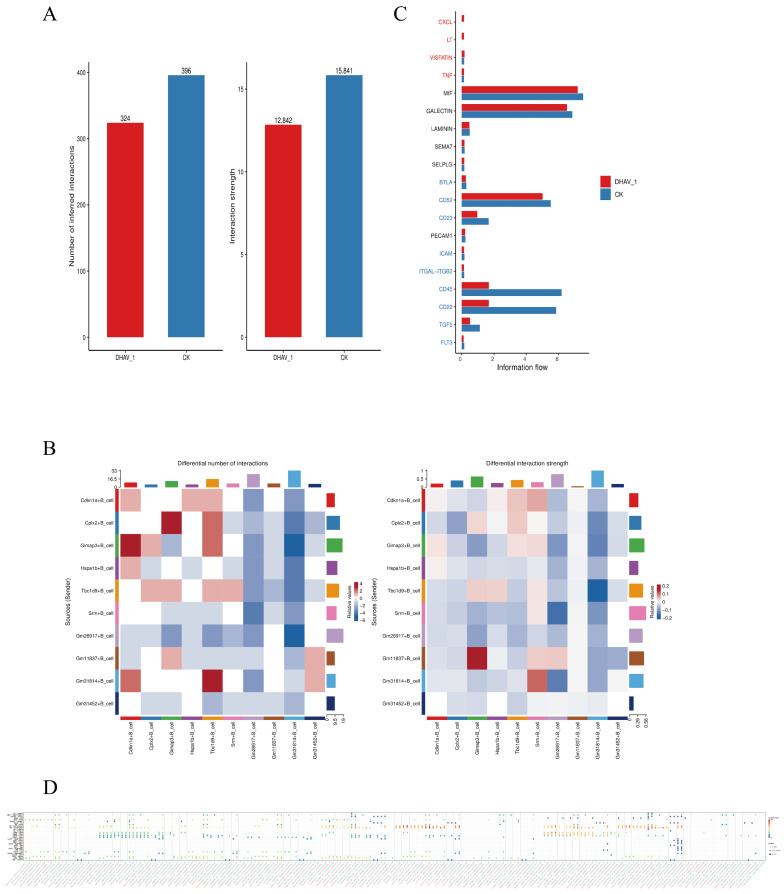
Analysis of Cell Communication Mechanisms Between the DHAV-1 and CK Groups. (**A**) Bar chart comparing the number and strength of cell–cell interactions. (**B**) Heatmap comparing the number and strength of cell–cell interactions. (**C**) Overall information flow comparison across the signaling pathways (Red indicates enrichment in the DHAV-1 group; blue indicates enrichment in the CK group). (**D**) Bubble chart depicting the relationships between the signaling pathways.

Analysis of cell–cell communication revealed that both the information and intensity metrics of intercellular communication in the DHAV-1 group were reduced compared to those in the CK group (Figure 3A). However, upon examining the interaction strength between the two groups, compared with the CK group, the intercellular communication intensity was reduced in Cplx2+B_cells, Gm11837+B_cells, Gm31814+B_cells, and Gm31452+B_cells. However, the signal intensity increased between the Gm26917+B_cells and Gm11837+B_cells (Figure 3B). These signaling pathways indicate that B cells play a crucial role in regulating the cell cycle, protein synthesis, and autophagy. Reduced signaling intensity is typically associated with impaired intercellular communication and immune dysregulation, including immune system disorders and infectious diseases.

Information flow refers to the transmission and exchange of genetic and other biological information within and between cells. As shown in Figure 3C, CXCL and LT expressions were enriched in the DHAV-1 group. Members of the CXCL family exhibit diverse functions with distinct roles in immune regulation [28], tumor progression, and inflammatory responses [29]. Lymphotoxin (LT) is a cytokine secreted by lymphocytes upon activation by antigens or mitogens [30] and in certain tumors and autoimmune diseases. Activation of the lymphotoxin-β receptor (LTβR, TNFRSF3) signaling pathway governs the responses controlling cell differentiation, growth, and death. These responses manifest in the formation and tissue architecture of peripheral lymphoid organs, dendritic cell homeostasis, liver regeneration, interferon responses to pathogens, and death of mucosal-derived cancer cells.

VISFATIN and TNF integrin subunits are upregulated, and visfatin participates in multiple biological processes and modulates the expression of related cytokines. This allows visfatin to regulate inflammation and apoptosis in RAW264.7 cells and mouse immune organs. Tumor Necrosis Factor (TNF) is extensively involved in multiple physiological and pathological processes, including inflammatory responses, cell proliferation, differentiation, apoptosis, and immunoregulation. The expression levels of MIF, galectin, CD52, CD45, and CD22 were downregulated. MIF participates in neuroinflammation by inducing the production of proinflammatory cytokines, thereby regulating neuronal survival and neuroplasticity [31]. It also modulates macrophage function in host defense by suppressing the anti-inflammatory effects of glucocorticoids [32]. It is currently considered the only cytokine known to negatively regulate the anti-inflammatory effects of glucocorticoids (GC) [33]. Galectins achieve this by regulating apoptotic signaling pathways and influencing tumor cell growth and multiple forms of cell death. They further modulate the functions of various immune cells, including T lymphocytes, macrophages, and dendritic cells, thereby participating in tumor-immune escape processes [34]. CD52 interacts with sialic acid-binding immunoglobulin-like agglutinin 10 (Siglec-10), significantly inhibiting T cell proliferation and activation [35]. CD45 plays a crucial role in viral defense; however, certain viruses exploit CD45 to evade immune recognition and clearance. Notably, srm+B cells and Gm31814+B_cells were enriched in the signaling pathways (Figure 3D), suggesting potential candidate cell subsets for subsequent therapeutic target exploration. Appropriately restoring the immune response of the infected host could achieve corresponding therapeutic goals.

### 3.4. Analysis of B-Cell and CDR3 Diversity

**Figure 4:**
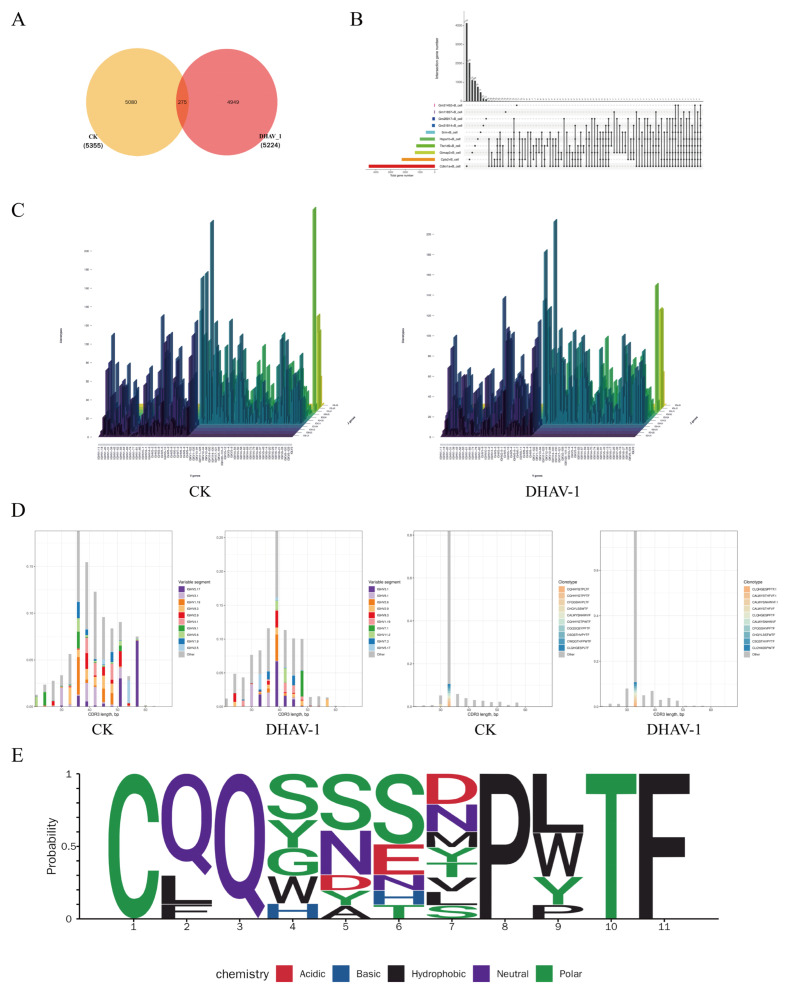
Differential Analysis of VDJ and CDR3 Between the DHAV-1 and CK Groups. (**A**) Shared clonotype Venn diagram. (**B**) Expression levels of clonotypes in different B cell subpopulations. (**C**) Frequency distribution of V-J gene combination clonotypes in each sample, visualized as a three-dimensional bar chart. The X-axis represents the V genes, the Y-axis represents the J genes, and the Z-axis represents the number of corresponding clonotypes. (**D**) Distribution map of V gene composition across different CDR3 lengths. Distribution of CDR3 amino acid sequence types across varying CDR3 amino acid sequence lengths for each sample. (**E**) Sequence logo generated by sorting CDR3 sequences by frequency and selecting the corresponding amino acid sequences.

The immune response mechanisms triggered by DHAV-1 recognition remain unclear. By constructing a mouse BCR library for clonal profiling (Figure 4A), we identified 5224 distinct clonal types in the DHAV-1 group, including 275 clones shared with the CK group. The expression levels of clonotypes in the DHAV-1 group were higher than those in the CK group. The Cdkn1a+ B cell subtype exhibited the highest gene expression level, indicating its crucial role in DHAV-1 recognition and subsequent immune responses (Figure 4B). The BCR comprises variable and constant regions, with high diversity arising from V(D)J recombination. In the heavy chain (H chain) variable region, DHAV-1 group mice primarily utilized genes from the IGHV1, IGHV2, and IGHV5 families, with the IGHV1 family being particularly prominent in this regard. In the light chain (L chain), gene fragments such as IGKV3 and IGKV4 were frequently observed.

As shown in Figure 4C, the analysis revealed the most common V-J gene pairing in the DHAV-1 group. The IGHV3-1/IGHJ3 and IGHV2-2/IGHJ4 combinations were predominant in the H chain, whereas IGKV1-117/IGKJ1 and IGKV10-96/IGKJ1 were the primary pairing combinations in the L chain. CDR3 is a highly variable segment of the BCR located within the variable region (V region), which is crucial for antigen recognition and binding. BCRs that share the same CDR3 amino acid sequence originate from the same clone. As shown in Figure 4D, although high-frequency clonal types remained relatively stable, the amino acid sequence length in the CDR3 region of the H chain in the DHAV-1 group tended to be shifted toward shorter lengths than that in the CK group. Moreover, the diversity of H chain clonal types in the DHAV-1 group exhibited a decreasing trend compared to that in the CK group. This phenomenon may indicate that under stimulation by DHAV-1 antigens, B cells undergo clonal selection and expansion, with dominant clones gaining a preponderant position, thereby leading to a reduction in overall clonal diversity.

According to the amino acid sequence logo analysis (Figure 4E), the cysteine at position 1 and glutamine at position 3 are both highly conserved sites. These sites predominantly feature polar and neutral amino acids. This distribution ensures the stability and functional persistence of immunoglobulins within the body. Positions 4–7 exhibit diverse amino acid properties, constituting a highly variable region that serves as the specific binding domain. This region provides multiple binding sites to modulate protein affinity and adapt to the surface structures of different interacting proteins. The synergistic action of conserved and variable regions enables precise differentiation between multiple pathogens.

## 4. Discussion

Humoral immunity primarily functions through the production of specific antibodies during viral infections. In this study, we established a mouse model using inactivated DHAV-1 as the model viral antigen. On day 28 post-immunization, splenic B cells were isolated and sorted using magnetic bead separation. scRNA-seq was employed to analyze B cell gene expression profiles, decipher intercellular communication networks, and construct a BCR gene library. These efforts lay a crucial foundation for deepening our understanding of the transcriptional and BCR repertoire features associated with DHAV-1-induced B cell responses. B cell receptors possess specificity and diversity, playing crucial roles in immune defense, antigen presentation, cytokine production, and memory immune responses. This lays the foundation for designing specific vaccines.

By isolating and analyzing mouse splenocytes, this study identified various cell subpopulations, including B cells, T/NK cells, proliferating B cells, plasma cells, monocytes, and neutrophils. Based on the marker gene expression profiles, B cells were further subdivided into 10 distinct subsets. The elevated expression of Cdkn1a^+^ B cells in the DHAV-1 group compared to that in the CK group is highly consistent with the known function of Cdkn1a in regulating inflammatory homeostasis and suppressing excessive immune damage. This subpopulation may alleviate DHAV-1-induced hepatic inflammation and hemorrhagic lesions by downregulating the release of pro-inflammatory cytokines. Among the immunoglobulin heavy chain variable region (IGHV) genes, the IGHV1 family exhibited the highest clonal frequency, with IGHV1-81 being enriched in the DHAV-1 group. This suggests that IGHV1-81 may be part of the candidate BCR repertoire enriched post-immunization with inactivated DHAV-1 antigen, potentially contributing to the generation of antibody diversity. However, the potential role of IGHV1-81 in antigen binding (e.g., to DHAV-1 VP protein) has not been verified by epitope mapping, antibody functional assays, or structural modeling, and thus requires further experimental testing to confirm.

In this study, the upregulation of inflammation-related genes (S100a8, S100a9, and CAMP) at the transcript level suggests potential involvement in modulating immune cell recruitment and activation, which may contribute to the inflammatory response induced by inactivated DHAV-1 antigen. The overall reduction in intercellular communication intensity in the DHAV-1 group may reflect altered immune cell crosstalk in response to antigen stimulation. Furthermore, altered transcript levels of cytokines such as VISFATIN, MIF, and GALECTIN prompt possible associations with immune response-related pathways. Analysis of the CDR3 amino acid sequence diagram revealed a highly conserved cysteine at position 1 of the sequence. Cysteine, a key sulfur-containing amino acid, serves as an essential precursor molecule in poultry immune defense, antioxidant systems, cellular signaling, and gene expression. It regulates growth, development, and health status in poultry, may play a potential role, particularly in immune cell activation and antioxidant stress response [36]. Glutamine significantly downregulates the mRNA expression of LPS-induced proinflammatory factors (IL-6, IL-1β, TNF-α) in the liver, thereby alleviating hepatic inflammatory damage [37].

This study employed scRNA-seq to investigate immune cell interactions. Silencing of Gm26917+ B cells attenuates lipopolysaccharide-induced inflammation [13,38]. Additionally, CXCL and LT are upregulated following immunization, playing crucial roles in various physiological and pathological processes, including immune regulation, inflammatory responses, and tumor progression. In contrast to DHAV-3, which modulates immune responses through signaling pathways and gene expression regulation [14], DHAV-1 directly participates in inflammatory responses and immune tissue development. DHAV-1 typically exhibits a greater propensity to invade immune tissues, directly triggering tissue damage-related inflammatory pathways [39,40]. For DHAV-3, the host tends to establish specific immunity through finely tuned signaling pathways and gene expression regulation [41]. This discrepancy highlights the diversity of immune responses induced by different viral antigens—even within the same genus—and underscores the value of DHAV-1 as a model for studying antigen-specific immune mechanisms.

Due to species-specific differences between mice and ducks, findings from this mouse model are limited to the humoral immune response to inactivated viral antigens in mice and cannot be extrapolated to duck-related immunological studies or natural DHAV-1 infection in ducks. Nevertheless, this study provides important insights into the fundamental mechanisms of B-cell responses induced by inactivated viral antigens. By systematically characterizing B cell subset dynamics, intercellular communication networks, and B cell receptor repertoire features in mice immunized with DHAV-1 as a model antigen, our findings enrich the understanding of the multifunctional role of B cells in virus antigen-induced immune responses and lay the foundation for further exploration of universal humoral immune mechanisms against antiviral antigens. The core value of the mouse model lies in its ability to characterize the transcriptional and clonal dynamics of B cells induced by inactivated viral antigens within a strictly controlled experimental system, providing a preliminary framework for understanding the fundamental principles of humoral immune responses to viral antigens. Inactivated DHAV-1, with its stable immunogenicity and well-defined molecular structure, serves as an ideal model antigen for elucidating these processes, independent of confounding factors such as viral replication or species-specific pathogenicity.

The mouse immune system has a well-defined genetic background, with minimal variation in immune responses among animals from the same batch, making it a widely recognized, robust, and reliable experimental model. It also features versatile single-cell sequencing and BCR library technologies, enabling the analysis of molecular interactions between DHAV-1 and the immune system and facilitating rapid BCR-related research. In contrast, the duckling model lacks a fully developed BCR library and corresponding immunological reagents and technical systems, making it difficult to achieve research at the same level of maturity. In contrast, while ducklings serve as natural hosts for DHAV-1 and closely resemble clinical infection scenarios, they present challenges such as high genetic background heterogeneity and significant individual variation. Furthermore, incomplete annotation of their BCR genes, lack of specific immunoassay reagents, and absence of mature high-throughput research technologies hinder the detailed investigation of B-cell immune regulatory mechanisms required for this study. Therefore, considering experimental reliability, technical feasibility, and research depth requirements, the mouse model enables efficient and precise analysis of the interaction patterns between DHAV-1 and B-cell immunity. However, this also implies that our findings reflect mammalian rather than avian immune characteristics and are applicable only to understanding the humoral immune mechanisms of mice against inactivated viral antigens. The core value of using a mouse model lies in its ability to precisely reveal the fundamental principles of B-cell responses induced by viral antigens, which is the primary focus of this study.

Currently, the cross-reactivity between high-titer antibodies and VP proteins in mouse models confirms their utility for the rapid screening of DHAV-1 vaccine immunogenicity [42]. Furthermore, Sun et al. validated the neutralizing antibodies against respiratory syncytial virus using mouse models [43]. Similarly, mouse models have successfully identified neutralizing antibodies against the SFTSV [44]. These studies confirm that mouse models are suitable for analyzing antibody responses induced by inactivated viral antigens at the fundamental immunological level. Furthermore, inactivated DHAV-1 possesses a well-defined molecular structure and stable immunogenicity. As a model antigen, it eliminates confounding factors such as viral replication and pathogenicity, allowing focused investigation of core humoral immune response processes. Therefore, employing inactivated DHAV-1 as a model antigen, combined with the well-established immunological research framework of mouse models, enables efficient characterization of B-cell response features and BCR repertoire dynamics induced by viral antigens. Due to the high cost of the 10× Genomics platform, this study carries the risk of an insufficient sample size and inadequate data representativeness. Therefore, we meticulously designed an experimental protocol to minimize systematic variations between samples and enhance the consistency and stability of the results. This study provides a preliminary theoretical foundation for characterizing BCR-related transcriptional and clonal features in humoral immunity against DHAV-1 in mice. Furthermore, the core objective of subsequent research is to conduct in-depth investigations into antibody function and its mechanisms of action using single-cell sequencing and BCR analysis. This will further elucidate the pivotal role of B cells in humoral immunity, validate relevant sites in vivo, and identify superior antibody subtypes with core functional significance in the future.

## 5. Conclusions

This study systematically analyzed the immunological characteristics and BCR repertoire of mice immunized with inactivated DHAV-1, revealing for the first time the response patterns of B cell subsets induced by this model antigen in a mouse model. It identified the molecular features of B-cell-associated inflammatory and immune responses following DHAV-1 immunization. This study offers preliminary transcriptomic and BCR repertoire insights into B-cell responses following DHAV-1 immunization in mice, providing a groundwork for future mechanistic investigations into mammalian humoral immune responses to inactivated viral antigens. Due to immunological differences between species, these findings only support the formulation of immunological hypotheses for duck-derived inactivated DHAV-1 antigens, and subsequent validation in natural host duckling models with live virus infection is needed to fully clarify the genuine immune mechanisms of host resistance to DHAV-1.

## Data Availability

The data supporting the findings of this study are contained within the article. The data and materials that support the findings of this study are available from the corresponding author upon reasonable requests.
